# A novel missense variant of the *GNAI3* gene and recognisable morphological characteristics of the mandibula in ARCND1

**DOI:** 10.1038/s10038-021-00915-z

**Published:** 2021-03-15

**Authors:** Kumiko Yanagi, Noriko Morimoto, Manami Iso, Yukimi Abe, Kohji Okamura, Tomoo Nakamura, Yoichi Matsubara, Tadashi Kaname

**Affiliations:** 1 Department of Genome Medicine, National Center for Child Health and Development, Setagaya, Tokyo Japan; 2 Division of Otolaryngology, National Center for Child Health and Development, Setagaya, Tokyo Japan; 3 Department of Pharmacology, National Center for Child Health and Development, Setagaya, Tokyo Japan; 4 Department of Systems BioMedicine, National Center for Child Health and Development, Setagaya, Tokyo Japan; 5 Division of General Pediatrics & Interdisciplinary Medicine, National Center for Child Health and Development, Setagaya, Tokyo Japan; 6 National Center for Child Health and Development, Setagaya, Tokyo Japan

**Keywords:** Genetic testing, Development

## Abstract

Auriculocondylar syndrome (ARCND) is an autosomal monogenic disorder characterised by external ear abnormalities and micrognathia due to hypoplasia of the mandibular rami, condyle and coronoid process. Genetically, three subtypes of ARCND (ARCND1, ARCND2 and ARCND3) have been reported. To date, five pathogenic variants of *GNAI3* have been reported in ARCND1 patients. Here, we report a novel variant of *GNAI3* (NM_006496:c.807C>A:p.(Asn269Lys)) in a Japanese girl with micrognathia using trio-based whole exome sequencing analysis. The *GNAI3* gene encodes a heterotrimeric guanine nucleotide-binding protein. The novel variant locates the guanine nucleotide-binding site, and the substitution was predicted to interfere with guanine nucleotide-binding by in silico structural analysis. Three-dimensional computer tomography scan, or cephalogram, displayed severely hypoplastic mandibular rami and fusion to the medial and lateral pterygoid plates, which have been recognised in other ARCND1 patients, but have not been described in ARCND2 and ARCND3, suggesting that these may be distinguishable features in ARCND1.

## Introduction

Auriculocondylar syndrome (ARCND) is a rare autosomal dominant or recessive disorder characterised by recognisable malformation of the ears, known as question-mark ears, and micrognathia involving hypoplasia of the mandibular rami, condyle and coronoid process. These orofacial malformations lead to respiratory difficulties, including apnoea. Feeding and speech difficulties due to ankylosis of the temporomandibular joints often become the chief complaints [1].

ARCND is genetically classified into three subtypes, ARCND1 (MIM#602483), ARCND2 (MIM #614669) and ARCND3 (MIM #615706), whose causative genes are *GNAI3* [1–6], *PLCB4* [2, 3, 7–9] and *EDN1* [10], respectively. *GNAI3* encodes guanine nucleotide-binding protein subunit α (Gαi3), a member of the heterotrimeric guanine nucleotide-binding proteins (G proteins). Gαi3 (NP_006487.1) has five guanine nucleotide-binding sites, G1–G5 boxes, in the GTP catalytic domain. The amino acid sequences in the G boxes are essential to the binding of guanine nucleotides [11]. To date, five pathogenic variants have been reported: three variants in the G1 box (p.Gly40Arg, p.Gly45Val and p.Ser47Arg), one in the G4 box (p.Asn269Tyr) and one which locates just one amino acid outside of the G1 box (p.Thr48Asn) [2, 6, 10].

Here, we present a novel variant in the G4 box of Gαi3 found in a Japanese girl tentatively diagnosed with severe micrognathia and describe recognisable morphological characteristics of the mandibula specific in ARCND1.

## Case report

**Fig. 1:**
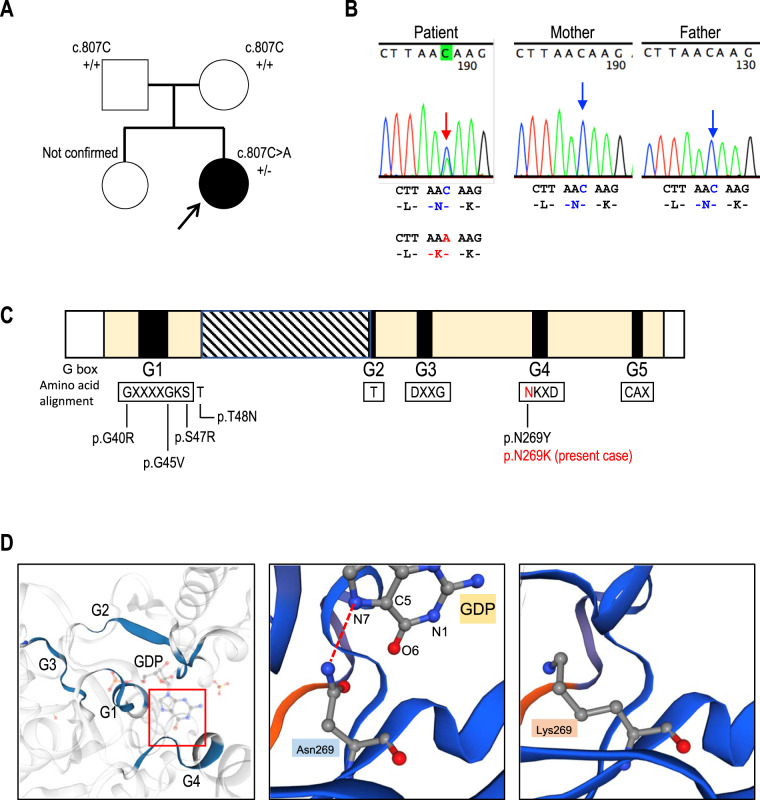
Pedigree and genetic analysis. **A** Pedigree of the family. There was no family history. The proband has an elder sister with normal appearance. The mother has no history of trauma or exposure to any known teratogens or intake of any medication during pregnancy. **B** Electropherograms of Sanger sequence. The heterozygous *de novo* variant in the patient, c.807C>A (*GNAI3*:NM_006496), is indicated by the red arrow (left panel). The same nucleotide in her parents is indicated by the blue arrow (middle and right panel). The amino acids translated from the DNA sequences are presented under each electropherogram. **C** Schematic diagram of Gαi3 (NP_006487.1). Five guanine nucleotide-binding site (G1–G5, black box) are located within GTP catalytic domains (yellow box). The catalytic domain is divided by an α helical domain (diagonal stripes). Consensus amino acid residues of the nucleotide-binding sites are boxed and indicated under each bonding site. Gαi3 variants from the literature are indicated in black and the novel variant identified in this case is indicated in red. **D** In silico three-dimensional views of the Gαi3. Wild-type Gαi3 was obtained from a database of annotated 3D structures generated by SWISS-MODEL (UniProtKB AC; P08754, PDB ID; 4g5o). Regions of the guanine nucleotide-binding site, G1–G4, are indicated in blue. The GDP-binding region concerning Asn269 is indicated by the red square (left panel). The side chain of Asn269 in wild-type Gαi3 makes a hydrogen bond to the N7 atom of the GDP (red broken line, middle panel). Lysin is a charged amino acid with a longer carbon skeleton compared to asparagine. The ε amino group of Lys269 is facing the opposite side (arrow), which would affect the hydrogen bond to GDP (right panel, GDP is not shown). The structure of the Gαi3 variant (p.Asn269Lys) was constructed based on the guanine nucleotide-binding protein G(k) subunit alpha by the SWISS-MODEL server homology modelling pipeline

**Fig. 2:**
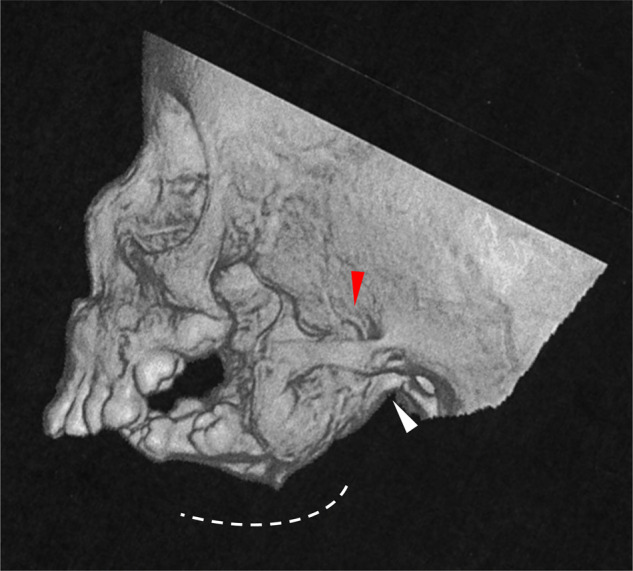
Three-dimensional computed tomography (3D-CT) image of the patient. Mandibular condyle agenesis and excessively hypoplastic rami and processes can be observed. The possible positions of the mandibular jaw (white arrowhead) and process (red arrowhead) are indicated. The mandibular angle is unclear

The proband was a 2-year-old Japanese girl born to healthy non-consanguineous parents (Fig. 1A). She presented severe mandibular hypoplasia and a rounded facial appearance with prominent cheeks. External ear malformation (question-mark ears) was observed. Oral malformations, such as microstomia, lobular hypoplastic tongue with soft-tissue projections, cleft palate with hypoplastic soft palate and ankylosis of the temporomandibular joints, were also present (Supplementary Figure). She also had difficulties with feeding and speech articulation. Meatal stenosis, malformation of the internal ear (semicircular and cochlear duct) and moderate sensorineural hearing loss were seen. Tracheostomy was performed due to upper airway obstruction. Three-dimensional computed tomography (3D-CT) showed mandibular condyle agenesis, excessively short rami and retrognathia (Fig. 2). The mandibular rami were fused with medial and lateral pterygoid plates. Both her mandibular angles were unclear. The intracranial structure, nasal cavity, orbital cavity and maxilla were normal. No other general malformations, growth retardation or developmental delay was observed. The clinical findings for this case are summarised in Table 1.

**Table 1:** Summary of clinical features and variants of patients with ARCND1 in present case and reported in literatures

Reference of Genetic analysis (case#)	Present report	Rieder et al. [2] (#S008)	Rieder et al. [2] (#S011)	Tavares et al. [6] (#Sp1)	Gordon et al. [3] (#Case7)	Tavares et al. [6] (#Sp2)	Tavares et al. [6] (#ACS1)
Reference of case report (case#)	Present report, *de novo*	Rieder et al. [2] (#S008), Familial case, Inherited from her father with mild hypoplasia	Erlich et al. [4] (#patient1), Familial case, Inherited from her mother with similar phenotype	Propst et al. [5], *de novo*	Gordon et al. [3] (#Case7), Familial case, Inherited from her father who have normal ears and mild hypoplastic of mandibula	Tavares et al. [6] (#Sp2), *de novo*	Guion-Almeida et al. [1] (#III-18), Familial case
Reference of 3D-CT images (case#)	Present report	Rieder et al. [2] (#S008)	Erlich et al. [4] (#patient1)	Propst et al. [5]	−	−	Passos-Bueno et al. [14] (#ACS)
Nucleotide (NM_006496.4)	c.807C>A	c.118G>C	c.118G>C	c.134G>T	c.141C>A	c.143C>A	c.805A>T
Amino acid (NP_006487.1)	p.N269K	p.Gly40Arg	p.Gly40Arg	p.Gly45Val	p.Ser47Arg	p.Thr48Asn	p.Asn269Tyr
Location (G box)	G4	G1	G1	G1	G1	G1	G4
Gender	Female	female	Female	Male	Female	Male	Female
Micrognathia	Severe	Severe	Severe	Severe	Asymmetric	Asymmetric	Severe
Round facial appearance	+	+	+	+	+	+	+
Prominent cheeks	+	+	+	+	+	+	+
Low-set ears	+	+	+	+	+	+	+
Malformed ears/question-mark ears	+	+	+	+	+	+	+
Auricular clefts	+	−	+	+	−	−	+
Overfolding of the superior helices	+	+	+	+	−	+	+
External auditory canals	Stenotic	NA	Narrowing	Stenotic	NA	NA	Atretic
Hearing loss	Moderate	NA	Mild to moderate	Conductive	−	+	+
Malformation of middle and inner ear	+	NA	−	+	NA	NA	NA
Microstomia	+	+	+	+	+	+	+
Glossoptosis	+	+	+	+	NA	+	+
Abnormality of tongue	Lobulated with lateral soft-tissue projections	lateral soft-tissue projections	Hypoplastic	+	NA	+	–
Cleft palate	–	NA	+	−	–	+	NE
Dysmorphic features of mandibular condyle and coronoid	+	+	Temporomandibular joints are not seen	Maldevelopment of coronoid processes	NA	NA	Condyle agenesis
Hypoplastic mandibular rami	Severe	Severe	Severe (agenesis)	Severe	NA	NA	Severe
Mandibular angle	Unrecognisable	Unrecognisable	Unrecognisable	Unrecognisable	NA	NA	Unrecognisable
Ankylosis of mandibular jaw	+	+	+	+	NA	NA	Severely limited
Fusion to medial and lateral pterygoid plates	+	+	+	NA	NA	NA	NA
Respiratory difficulties	+	+	+	+	NA	+	+
Feeding difficulties	+	NA	NA	+	NA	+	+
Speech articulation difficulties	+	NA	NA	NA	NA	NA	NA
Apnea	+	NA	+	+	NA	+	NA
Tracheotomy	+	+	+	+	NA	NA	+
Gastrostomy tubes	+	NA	+	NA	NA	NA	+

Clinical symptom is only provided for the proband of family case

*+* Present, − absence, *NE* not evaluated, *NA* not reported in literatures

### Genetic and protein structural analyses

After obtaining written informed consent from her parents, whole exome sequencing (WES) analysis was performed in the patient and her parents (see Supplementary Methods) [12]. By trio-based filtering, we identified a novel *de novo* non-synonymous variant in exon 7 of *GNAI3* (NM_006496.4:c.807C>A:p.(Asn269Lys)). The variant was confirmed by Sanger sequencing (Fig. 1B). It was not found in the ExAC, gnomAD, 1000 genome or the inhouse database of WES from over 4000 Japanese individuals. In silico prediction programmes, such as PolyPhen2 and SIFT, estimated that the *GNAI3* novel variant was damaging. Asn269 within the G4 box domain of the Gαi3 forms a hydrogen bond with the N7 atom of the guanine moiety [13]. In silico structural analysis reveals that the substitution of Asn269 to Lys is expected to disrupt the hydrogen bond, which may interfere downstream of Gαi3 signal (Fig. 1D). According to the ACMG guidelines, the novel variant was classified as likely pathogenic (strong, PS2; moderate, PM2 and PM5; supporting, PP2 and PP3).

## Discussion

We report a patient with severe mandibular hypoplasia, external ear malformation and a rounded face with prominent cheeks. Trio-based WES analysis revealed a novel missense variant of Gαi3, p.(Asn269Lys), which is predicted disruption of the hydrogen bond to the N7 atom of the guanine moiety. One patient with p.Asn269Tyr, which was different substitution to Tyr, was reported [6]. The N7 seems to be backbone of tryptophane, therefore hydrophobic interaction might be likely and easy disrupted by substitution of Lysine, supporting that mandibular hypoplasia phenotype in our patient (Supplementary Fig. 1) is more severe than in the patient with p.Asn269Tyr [6]. Her head 3D-CT showed agenesis of the mandibular condyle, retrognathia and an excessively short mandibular rami, which were fused with the medial and lateral pterygoid plates.

The orofacial appearance is similar among cases of ARCND1 (Table 1; for photographs, refer to original article) [1–6]. We investigated and compared the appearance of the mandibular area among cases of ARCND1 [2, 4, 5, 14], ARCND2 [2, 3, 7, 8]　and ARCND3 [10] using 3D-CT images or radiological images. Anatomically, the distal region of the mandibula consists of the mandibular condyle, the coronoid process and the mandibular rami. The appearance in 3D-CT or radiological images in such mandibula are similar among ARCND1 cases. In our case, there was a lack of clarity of the mandibular angle due to the excess hypoplasia of the distal region and fusion between the mandibular rami and the pterygoid plate, which are common in ARCND1 patients (Table 1). In contrast, a clear mandibular angle and mild hypoplastic short mandibula are observed in patients with ARCND2 and ARCND3 (Supplementary Table S1). In addition, there is no description of fusion between the mandibular rami and the pterygoid plate in them (Supplementary Table S1). Although the obtained images from the literature were limited, these findings might be distinguishable features of ARCND1 from ARCND2 and ARCND3.

The development of the mandibula is uniquely regulated during embryogenesis and is also affected by postnatal food preference [15]. Dlx5/Dlx6 knockout mice show a hypoplastic mandibula similar to that found in ARCND [16]. Gnai3, Plcb4 and Edn1 act as upstream molecules in the endotherin-Dlx5/Dlx6 signalling pathway, and dysregulation of the pathway is involved in the pathogenicity of ARCND [2, 6, 17]. However, the mandibular hypoplasia of ARCND1 is much more severe than that of ARCND2 and ARCND3 (Table 1 and Supplementary Table S1). Another pathway may support such severity. It is interesting that both the mandibular condyle and the pterygoid process undergo cartilage and membrane ossification during their development and growth [18]. In mouse embryos, SRY-box 9 (Sox 9) was found to be first expressed in chondrocytes in the cranial base prior to expression of type X collagen at E14.5 and in chondrocytes of the condylar cartilage at E15.5 [18]. Both overexpression and knockout of Sox9 lead to dramatic inhibition of chondrocyte proliferation and terminal differentiation, which results in false endochondral-like ossification of these regions [19]. Sox9 is regulated by Gnai3 via PKA and cAMP [20]. Gαi3 variants could also affect the SOX9 expression during embryogenesis, possibly causing the severity.

### Web resources

Database. GnomAD: https://gnomad.broadinstitute.org. 1000 Genome. PolyPhen2: http://genetics.bwh.harvard.edu/pph2/. SIFT: http://provean.jcvi.org/genome_submit_2.php?species=human. ACMG: https://www.nature.com/gim/articles?type=acmg-standards-and-guidelines. SWISS-MODEL: https://swissmodel.expasy.org/interactive.

## Supplementary information

Supplemental Table 1

Supplemental Figure

Supplementary Methods

## Data Availability

The raw datasets are available from the corresponding author upon reasonable request.
