# Supplementary figures and images for: A novel missense variant of the GNAI3 gene and recognisable morphological characteristics of the mandibula in ARCND1
Source: J Hum Genet. 2021 Mar 15;66(10):1029–34. doi: 10.1038/s10038-021-00915-z (PMC8472909; doi:10.1038/s10038-021-00915-z)

## Slide 1
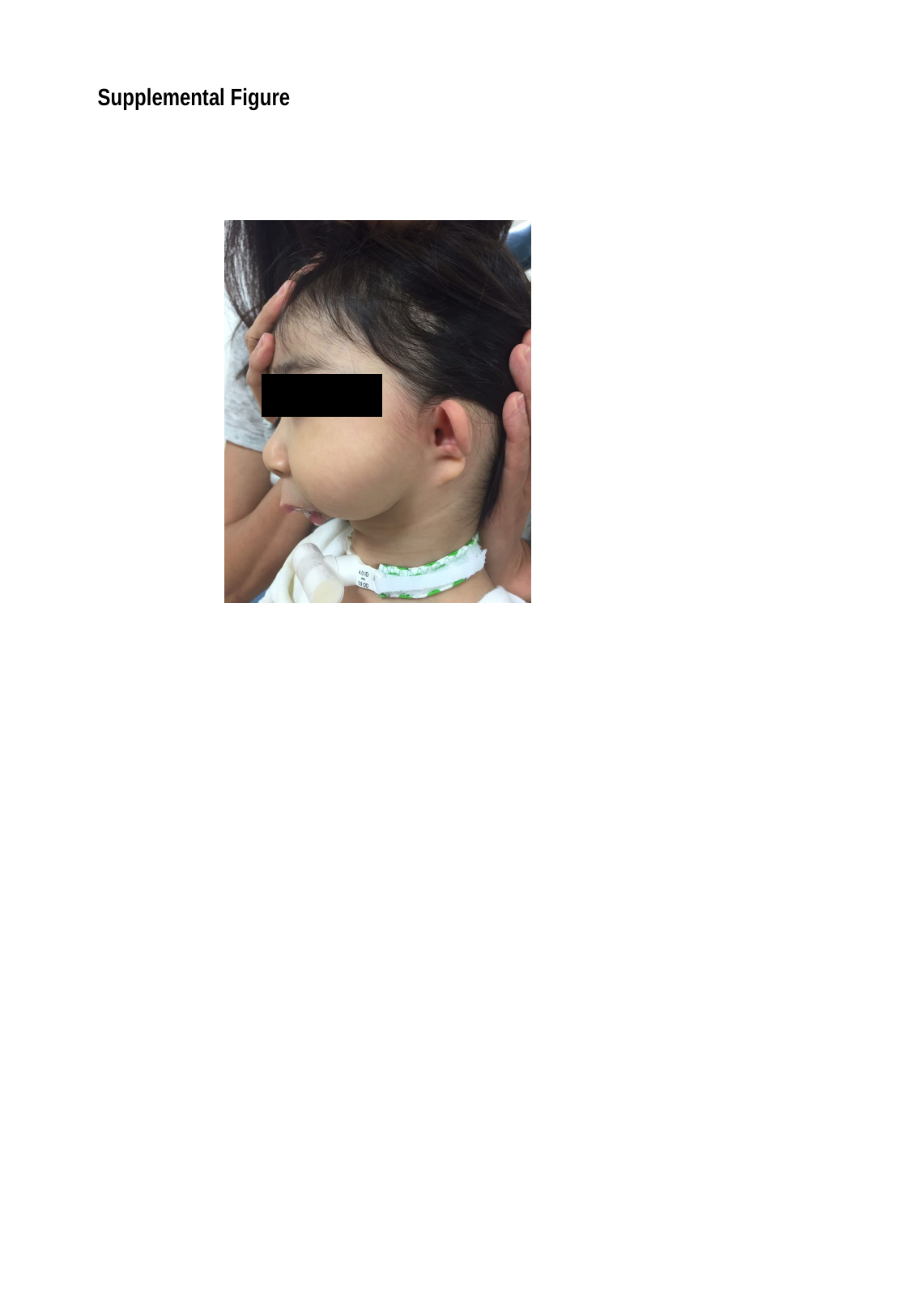

Supplemental Figure

Supplement: Supplementary file 2 — Supplemental Figure [file 10038_2021_915_MOESM2_ESM.pptx]
