# Supplementary material for: A novel missense variant of the GNAI3 gene and recognisable morphological characteristics of the mandibula in ARCND1
Source: J Hum Genet. 2021 Mar 15;66(10):1029–34. doi: 10.1038/s10038-021-00915-z (PMC8472909; doi:10.1038/s10038-021-00915-z)
Supplement: Supplementary file 3 — Supplementary Methods [file 10038_2021_915_MOESM3_ESM.docx]

Supplementary Methods

Genetic Analysis

Genomic DNA was extracted from the peripheral blood using a QIAamp DNA Mini Kit (Qiagen GmbH, Hilden, Germany). The sequence library was prepared using a Human All Exon V6 Kit (Agilent Technologies) and sequenced using a 2500 Illumina with 125-bp paired-end reads. Sequence reads were aligned to hg19 using a Burrows–Wheeler aligner. Variants were called GATK Unified Genotypes and annotated using ANNOVAR. Whole exome sequencing (WES) analysis was performed in the patient and her parents. Variants were narrowed based on allele frequency and assumed modes of inheritance, including autosomal dominant, autosomal recessive, X-linked and compound heterozygous mode. No pathogenic variants were noted in *TCOF1*, *POLR1C*, *POLR1D*, *PLCB4* and *EDN1*, which are identified in Treacher Collins syndrome (TCS1 MIM#154500, TCS2 MIM#613717 and TCS3 MIM#248390) and ARCND (ARCND2 MIM#614669 and ARCDN3 MIM# 615706). Segregated variants were finally confirmed by Sanger sequencing (ABI3130) with the primers 5′- TGGTGTTTGACTTATTTCCGTGA-3′ and 5′- CTGATATGGCACTCTTGACTTAGG-3′.

Database

GnomAD: https://gnomad.broadinstitute.org

1000 genome

PolyPhen2: http://genetics.bwh.harvard.edu/pph2/

SIFT: http://provean.jcvi.org/genome_submit_2.php?species=human

ACMG: <https://www.nature.com/gim/articles?type=acmg-standards-and-guidelines>

SWISS-MODEL: <https://swissmodel.expasy.org/interactive>
